# Comprehensive measurement of the prevalence of dementia in low- and middle-income countries: STRiDE methodology and its application in Indonesia and South Africa: commentary, Jain et al

**DOI:** 10.1192/bjo.2025.39

**Published:** 2025-06-11

**Authors:** Manisha Jain, Eef Hogervorst

**Affiliations:** School of Sport, Exercise and Health Sciences, Loughborough University, Loughborough, UK

**Keywords:** Dementia, prevalence estimates, risk and protective factors, low- to middle-income countries, epidemiology


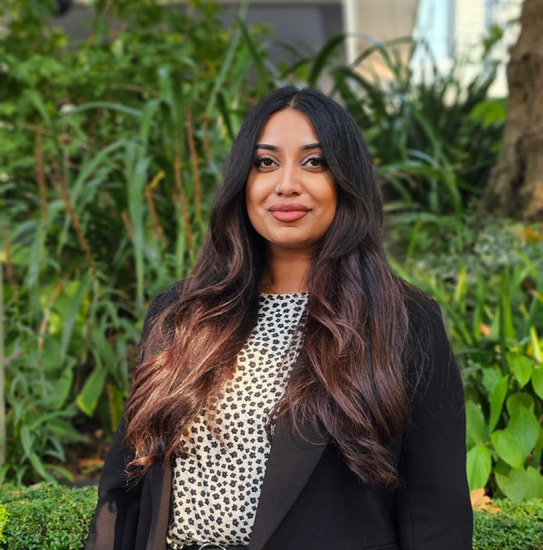



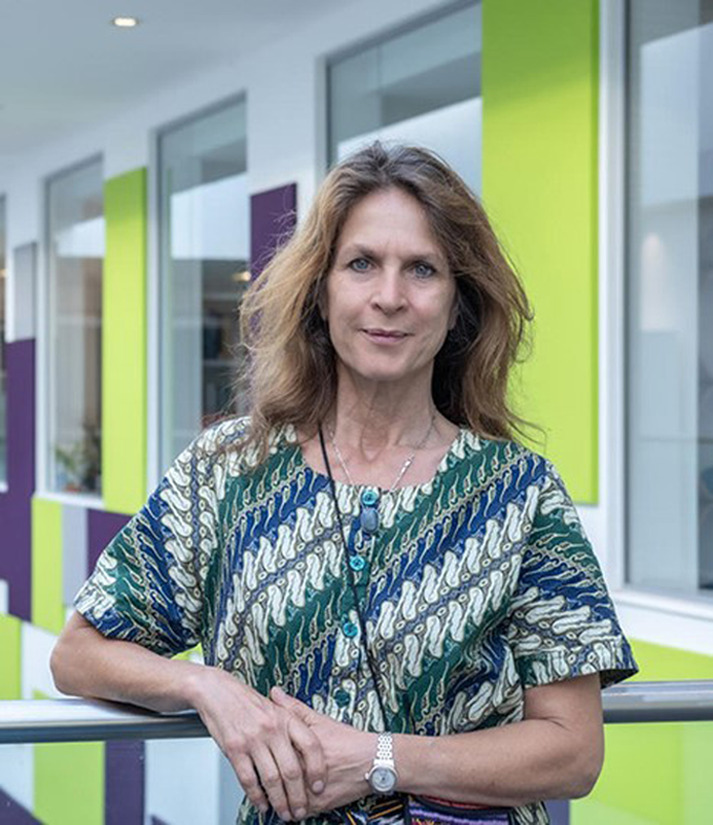


## Response

By 2050, two-thirds of the global older adult population could reside in low- to middle-income countries (LMICs), also home to an estimated 71% of the world’s dementia cases.^1^ This paper is a commentary on the published Indonesian dementia prevalence estimates developed using the Strengthening Responses to Dementia in Developing Countries (STRiDE) methodologies.^2^

The lack of national data for dementia prevalence in many LMICs means that estimates must rely on regional statistical modelling, less powerful than using local data.^2^ Current dementia prevalence estimates in Indonesia are >20% for the over-60s: two to three times higher than that of most countries included in the 2015 estimates from the World Alzheimer’s Report, e.g. Europe and the USA, with estimates between 4 and 9% for those aged over 60 years.^3–5^ STRiDE methodologies were used to estimate dementia prevalence in Indonesia.^2^ Recruitment of 2216 older people occurred in rural and urban Jakarta and North Sumatra, and who were assessed using the screening instruments 10/66 Short Dementia Diagnostic Schedule, Dementia Severity Rating Scale and Lawton Activities of Daily Living Scale. Nationwide estimates for dementia prevalence in this cohort were 27.9% in the over-60s, but only five individuals had a formal dementia diagnosis. These estimates are consistent with other Indonesian dementia prevalence estimates, ranging from 20 to 33%.^4–6^

A possible explanation for these high Indonesian dementia prevalence estimates could be that the diagnostic instruments used were too sensitive. Earlier research in 2006 showed lower estimated dementia prevalence in Indonesia (between 6 and 8%), which was similar to that in European countries using slightly different, perhaps more culturally adapted, dementia screening tests.^7^ The 10/66 instruments were applied in other LMICs and were reported to have good sensitivity for dementia.^8^ The prevalence in South Africa using these instruments was 12.5% in those aged over 60 years,^2^ almost double that in most Western countries and in our earlier Indonesian estimates.

In the STRiDE Indonesian study, men were less likely to have dementia, with an estimated prevalence of 21%, compared with women with 31%, and they were 2.5 times more likely to be literate,^2^ a protective factor for dementia. In our earlier study,^7^ dementia risk was double in rural areas, explained by education, poor health and, independently, older age. Gender was not a significant factor in these analyses, possibly because it was very closely associated with having had little education.^7,9^ Our earlier study showed the highest dementia estimates (16–21%) in Borobudur, a rural Javanese district, similar to the 2020 published estimates in that region for the over-60s (20%).^5,7^ This district had the oldest, but also poorest, population of those studied, who had little access to medical facilities and many without formal education.

While education affects cognitive reserve,^10^ it is also associated with reading,^11^ both shown to reduce dementia risk.^9^ According to national data, older Indonesian women were found to have lower education and engaged less in reading than older men.^12^ This lack of formal education could be associated with the delegation of roles within households and cultural attitudes towards girls. Lack of education, especially among older women in rural areas, could also cause difficulty in accurate dementia testing. Illiterate or poorly educated people may not be accustomed to formal examination environments, and anxiety surrounding their performance could affect the validity and reliability of cognitive assessments. The specificity of the 10/66 instrument was not reported, and could have been affected by such false positives.^2^

Many cognitive tests have been developed for Western, literate participants and must be cross-culturally adapted for LMICs, which may not have been carried out sufficiently.^2,7^ Farina et al suggested that the 10/66 Short Schedule was education fair, but their false-positive rate was 5.5%; perhaps rather than dementia, the algorithm detected mild cognitive impairment (MCI). In our previous studies, the culturally modified Mini-Mental Status Examination (mMMSE) and the modified Hopkins Verbal Learning Test (mHVLT) both showed good dementia and MCI sensitivity and specificity in both UK and Indonesian cohorts.^7,13^ The mHVLT had dementia cut-off scores similar to the UK sample and mMMSE had high sensitivity (100%), also with a similar cut-off. For better specificity, the mMMSE needed a lower cut-off score and was modified by education, unlike the mHVLT.

Despite these issues, modifiable risk factors for dementia and MCI are similar in both high-income countries (HICs) and LMICs.^14,15^ In earlier studies investigating dementia risk in Indonesia,^4^ risk reduction was associated with engagement in psychosocial activities such as physical activity and attending community activities.^9^ In urban Jakarta (but not in the rural cohorts), higher engagement in sports, better diets and frequent reading were associated with lower dementia risk,^9^ suggesting engagement in multiple, available protective activities. Nevertheless, for both urban and rural areas, attending community activities was associated with lower dementia risk,^9^ consistent with other Indonesian analyses.^4,16^ In STRiDE, only smoking was assessed as a lifestyle factor and was associated with increased dementia risk.^17^

Health-related dementia risk factors are the same in both HICs and LMICs, such as stroke and diabetes.^5,18,20^ These preventable morbidities (through medication, diet and exercise) and their high prevalence in Indonesia could also partly explain the high dementia prevalence. In our earlier study,^7^ the prevalence of these morbidities was lower but people may have also died before developing dementia. With a lack of specialist medical care in rural areas, such illnesses can go untreated or undetected. Reverse causality could also play a role, whereby individuals are potentially forgetting to seek medical treatment because of cognitive decline.

Modifiable factors, such as education, psychosocial activities and preventable or treatable morbidities, are involved in the compression of dementia risk, as demonstrated in the Compression of Needs model.^9^ This model suggests that good physical/material resources (e.g. healthy lifestyle, access to good healthcare and physical activity) and psychosocial resources (e.g. good educational attainment, continued education and skill development, social engagement and technology use), as well as supportive public health policies, can compress and reduce the likelihood of risk factors associated with dementia health morbidities that increase dementia risk (e.g. stroke, diabetes and cardiovascular disease). This model underpins prevention, allowing individuals to address risk and enact change to reduce this risk (Table 1).

**Table 1:** Factors explaining the high dementia prevalence in Indonesia

Factor category	Specific factor	Potential impact of dementia prevalence
Sociodemographic	Education and ability to read and write	Lower levels of formal education, as well as the inability to read or write, have been associated with a greater risk of dementia and can negatively impact cognitive reserve. The lack of education in early life is considered a risk factor for dementia.
	Sex	Women have a greater risk of dementia, and this could be further exacerbated in rural areas where women are less likely to gain a formal education compared with men. In Farina et al’s study, men were less likely to have dementia and were 2.5 times more likely to be literate.
Cognitive engagement	Reading	Engagement in frequent reading could build cognitive reserve and be protective against dementia.
Lifestyle	Physical activity	Physical inactivity is a risk factor for dementia. Engagement in physical activity reduces the risk of dementia and can also reduce the risk of health morbidities associated with greater dementia risk, such as cardiovascular disease or diabetes. However, there may be lifestyle differences between urban and rural Indonesian communities.
	Smoking	Smoking is a risk factor for dementia and is associated with an increased risk of health morbidities that can also increase dementia risk.
	Diet	Maintenance of a good, healthy diet could reduce dementia risk; however, there might be differences between urban and rural areas.
	Community activities	Engagement in community activities could maintain social connectedness, which may combat loneliness, a factor associated with greater dementia risk. Such activities could also provide cognitive stimulation and improve physical activity.
Health morbidities	Stroke	There is a strong association between stroke and vascular dementia, and individuals with poorer cardiovascular health may be at a greater risk. Smoking and physical inactivity can exacerbate the risk for both stroke and dementia.
	Diabetes	Diabetes is a risk factor for developing dementia.
Healthcare access	Lack of specialist medical care	This can lead to delays in diagnoses and treatments, which could further exacerbate dementia. Healthcare infrastructure in lower-middle-income countries, particularly in rural areas, is often underdeveloped.
Diagnostic tools	Sensitivity	If diagnostic tools are too sensitive, they may overestimate dementia prevalence and give results that are not truly representative. However, the 10/66 Short Schedule instrument used in Farina et al’s study has been reported to have good sensitivity for dementia. They indicated that it was education fair, and the algorithm might have detected milder cases rather than false positives.
	Specificity	The lack of specificity of diagnostic tools could lead to false positives, indicating dementia cases where there may not be any. Instruments should be cross-culturally adapted and validated for use in lower-middle-income countries, because these are often developed for Western, literate individuals.
	Testing anxiety	The anxiety around cognitive testing could lead to poorer performance on diagnostic tests due to unfamiliarity with testing environments, possibly resulting in false positives. This could be less frequent for individuals who have had a formal education and are familiar with such environments. The specificity of the 10/66 Short Schedule instrument in Farina et al’s study was not mentioned.

Overall, Farina et al have contributed to increasing evidence suggesting Indonesian dementia estimates of approximately 20% in older adult populations. Possible explanations for these high estimates thus lie in (a) the challenges in ethnic or cultural differences in adapting cognitive assessments to LMICs, (b) a lack of exposure to previous examinations and testing, (c) issues with specificity and false positives possibly related to inadequately adapted cognitive tests and (d) the role of a greater magnitude of potentially preventable risk and protective factors, such as the lack of available psychosocial engagement, education, stroke and diabetes (Table 1). There may also be contributions to dementia risk from non-assessed communicable (infectious) diseases, such as malaria and tuberculosis, which are still important factors in LMICs.

With the increasing migration of young people to urban areas, alternative care and prevention for dementia in vulnerable older people become more important. With many risk factors and few medical specialists, dementia in LMICs is perhaps not treated or prevented well. Such issues need to be addressed at policy levels, given that future estimates predict that the fastest increasing dementia rates will be from LMICs.
